# New Diagnostic Tools for Pulmonary Embolism Detection

**DOI:** 10.14797/mdcvj.1342

**Published:** 2024-05-16

**Authors:** Jacob Shapiro, Adam Reichard, Patrick E. Muck

**Affiliations:** 1 Good Samaritan Hospital, Cincinnati, Ohio, US; 2 Bethesda North Hospital, Cincinnati, Ohio, US

**Keywords:** pulmonary embolism, artificial intelligence, triage, venous thromboembolism, pulmonary embolism response team (PERT)

## Abstract

The presentation of pulmonary embolism (PE) varies from asymptomatic to life-threatening, and management involves multiple specialists. Timely diagnosis of PE is based on clinical presentation, D-dimer testing, and computed tomography pulmonary angiogram (CTPA), and assessment by a Pulmonary Embolism Response Team (PERT) is critical to management. Artificial intelligence (AI) technology plays a key role in the PE workflow with automated detection and flagging of suspected PE in CTPA imaging. HIPAA-compliant communication features of mobile and web-based applications may facilitate PERT workflow with immediate access to imaging, team activation, and real-time information sharing and collaboration. In this review, we describe contemporary diagnostic tools, specifically AI, that are important in the triage and diagnosis of PE.

## Overview of PE

Pulmonary embolism (PE) is a venous thromboembolic disease that represents the third most common cause of cardiovascular death after myocardial infarction (MI) and stroke, and many survivors experience functional impairment for several months after the event.^1,2^ Risk factors include prolonged immobility, postoperative state, cancer, pregnancy, obesity, older age, and certain genetic conditions.^3^ Computed tomography (CT) pulmonary angiography (CTPA) is the imaging method of choice to assess pulmonary vasculature in patients with suspected PE. With clinical evaluation, patients with PE are stratified into high-, moderate-, and low-risk categories.^1,3^ Right ventricular (RV) dysfunction on a CTPA or echocardiogram is used to risk stratify PE by detecting PE-related RV pressure overload and RV dilation that can result in left ventricular (LV) obstructive shock.^1,3^ Treatment focuses on hemodynamic and respiratory support, anticoagulation, and, in high-risk or severe cases, thrombolytics or embolectomies to dissolve or remove the thrombus.^3^

## Challenges in Awareness, Accurate Diagnosis, and Timely Treatment

Prompt recognition of acute PE and immediate initiation of anticoagulation and mechanical thrombectomy can reduce the risk of death.^4,5^ PE presentation can mimic a range of other conditions, and the most common cause of death from PE is failure to diagnose. While CTA is the imaging modality of choice to diagnose PE, its diagnosis is time-consuming and requires radiology expertise.^4^ Over the last two decades, the use of CTA has increased over 27-fold, increasing the workload of providers and radiologists and further complicating the PE workflow. AI trained for PE detection and RV/LV measurement paired with real-time collaboration tools is well-suited to address these issues.^4^

## Impact of Use of AI in Treatment of Ischemic Stroke

Artificial intelligence (AI) in health care has become an essential radiological tool for detecting critical findings on computed tomography (CT) and can alleviate workloads while augmenting clinical practice.^6,7^ It has the potential not only to decrease errors, increase efficiency, and optimize consistency but also to evaluate vast amounts of real-time data for potential trends that can be used to improve processes. In the diagnosis and workflow of conditions like PE, AI is positioned to play a critical role.^7^ In fact, the impact of AI in the treatment of other time-sensitive diseases has been illustrated very clearly. There is perhaps no more time-sensitive illness in medicine than acute ischemic cerebral stroke, where the typical patient loses 1.9 million neurons for each minute that stroke is left untreated.^8^

The post-implementation impact of an AI-based triage and care coordination system has led to an accelerated time to treatment, a reduction in hospital length of stay (LOS), and better clinical outcomes.

An analysis of 86 stroke patients pre-AI implementation (average age of 68.53 ± 13.13 years, 40.7% female) and 102 stroke patients post-AI implementation (average age of 69.87 ± 15.75 years, 43.1% women) was performed at a large comprehensive stroke center (CSC). Following the implementation of the software, the mean door to puncture time interval within the CSC significantly improved by 86.7 min (206.6 versus 119.9 min; *P* < .001); significant improvements were also noted in the rate of reperfusion (modified Thrombolysis in Cerebral Infarction 2B-3) for patients in the post-AI population (*P* = .036).^9^

Additionally, AI can impact time to transfer and length of stay. Hassan and colleagues compared transfer time for all large vessel occlusion (LVO) transfer patients from a single Primary Stroke Center (PSC) to a CSC prior to and after incorporating AI Software (Viz.ai LVO). Using a prospectively collected stroke database at a CSC, demographics, modified Rankin Score (mRS) at discharge, mortality rate at discharge, hospital LOS, and neurological ICU were examined. There were a total of 43 patients during the study period (median age 72.0 ± 12.54 yrs, 51.16% women), with analysis of 28 patients from the pre-AI software (median age 73.5 ± 12.28 yrs, 46.4% women) and 15 patients from the post-AI software (median age 70.0 ± 13.29 yrs, 60% women). Following implementation of AI software, median CTA time at PSC to door-in at CSC was significantly reduced by an average of 22.5 min. (132.5 min versus 110 min; *P* = .0470). Hassan et al. reviewed their early experience utilizing AI. This research revealed significant reduction in transfer times and LOS in a hub and spoke model. The median LOS from admission to discharge and LOS in the neuro-ICU shows that the overall stay was considerably shortened in the post-AI software population (9 vs 7 days, *P* = .124) as well as in the neuro-ICU (5.5 vs 3 days; *P* = .00086]), pre- and post-AI software, respectively.^10^

An impact in clinical outcomes has also been noted in the use of AI in stroke treatment. A retrospective analysis of a prospectively maintained database was done for patients who presented to a stroke center currently utilizing Viz.ai LVO and who underwent endovascular thrombectomy following transfer for LVO stroke between July 2018 and March 2020. Time intervals and clinical outcomes were compared for 55 patients divided into pre- and post-Viz cohorts. Both cohorts were similar in terms of gender, age, proportion receiving intravenous tissue-type plasminogen activator, and proportion with revascularization of thrombolysis in cerebral infarction (TICI) classification > 2B. The presenting scores for National Institutes of Health Stroke Scale (NIHSS) and pre-stroke mRS were not statistically different. The median initial door-to-international normalized ratio notification was significantly faster in the post-Viz cohort (21.5 min vs 36 min; *P* = .02). The median initial door-to-puncture time interval was 20 min shorter in the post-Viz cohort, but this was not statistically significant (*P* = .20). The 5-day NIHSS and discharge mRS were both significantly lower in the post-Viz cohort (*P* = .02 and *P* = .03, respectively). The median 90-day mRS scores were also significantly lower post-Viz implementation, although a similar proportion received a good outcome (mRS score ≤ 2; *P* = .02 and *P* = .42, respectively).^11^

## AI-assisted Diagnosis and Treatment Optimization of PE

Imaging-focused AI algorithms designed to evaluate and detect PE on CTA have a high level of accuracy.^4^ In a matter of seconds, an AI algorithm evaluates a CTPA to quickly identify PE, including the size and location of an embolus.^7^ Guidelines acknowledge that an RV/LV ratio > 0.9 is a marker of CTPA-based RV dysfunction and right heart strain caused by the PE, but the accuracy of reported measurements is inconsistent.^3^ An AI platform that provides automated RV/LV ratio measurements can alleviate the need for echocardiography and allow for more efficient risk stratification.^3^

Fragmented pathways and lack of streamlined communication can further delay treatment decisions and negatively impact patient outcomes.^12,13^ With AI detection and automated PE alerts, care teams are able to simultaneously access crucial patient information, enabling timely assessment and treatment decisions that may improve a patient’s chance of survival.^14^ Clinicians can view CTA scans with axial image scrolling, manipulate window and level settings, and perform 3-dimensional anatomical rotations seamlessly on their phones, PACS workstation, or on an AI platform web portal. Integration of in-app communication and team activations reduces patient wait times for evaluation and hospital LOS.^12,15^ The urgency in diagnosing and treating PE, particularly in high-risk patients, presents a significant opportunity to streamline workflows similar to the guidelines established for MI and stroke.^16,17^

## Unique and Unmet Challenges Associated with PE

### Awareness, Detection, and Diagnosis

Often referred to as the “great masquerader,” PE can be extremely challenging to recognize and diagnose (Table 1).^18^ Patients with PE can present with symptoms that mimic acute MI, heart failure, syncope from arrhythmia, pneumonia, flu, asthma, panic attack, depression, or any other number of medical conditions. Establishing the diagnosis of PE first requires that the clinician include it in the differential diagnosis, yet clinicians often do not even consider PE as a potential cause of a patient’s symptoms.

**Table 1:** Unique and unmet challenges associated with pulmonary embolism.


Awareness, detection, and diagnosis Rapid notification and mobilization of the institutional PERT Risk stratification (integration of all data to estimate relative risk of morbidity/mortality Determination of optimal therapy Monitoring progress during/after intervention and establishing disposition Ascertaining risk of long-term consequences Expansion of evidence base


AI can play a valuable role in expediting diagnosis: AI programs can integrate clinical and historical information obtained from the chart and the clinician, and algorithms enhanced by machine learning (ML) can rapidly assess the probability of PE as a diagnosis. Imaging algorithms for CTPA scans, perfected and validated by AI companies, are remarkably accurate in establishing the presence of PE. What’s more, the analysis that establishes the diagnosis occurs in the background, virtually simultaneously with image acquisition. AI algorithms provide information regarding the size, location, and other aspects of the embolus. They also hold the potential to define the various components and age of the thrombus and the overall thrombus burden. AI programs approved by the US Food and Drug Administration also can quantify CT-derived RV/LV ratio more accurately and consistently than measurements made by individual physicians. Integration of echocardiographic findings, interpreted by AI algorithms based on ML, can further enhance diagnostic accuracy for detecting PE. Parameters from the echocardiogram include RV/LV ratio, RV overload/strain, underfilling of the LV, presence of a dilated pulmonary artery, patent foramen ovale, and other relevant cardiac pathology, all of which can influence therapeutic decision-making. By rapidly identifying PE, the decision-making clinicians and/or the PE response team (PERT) can be quickly notified, even before a radiologist interprets the scan.

It is important to note that AI not only facilitates the diagnosis of PE but also avoids missing the diagnosis. AI algorithms have been shown to be more sensitive and accurate than humans in detecting PE. This is a reflection of the consistency and systematic nature of the automated interpretations based on ML.

### Rapid Notification and Mobilization of Institutional PERT

**Figure 1:**
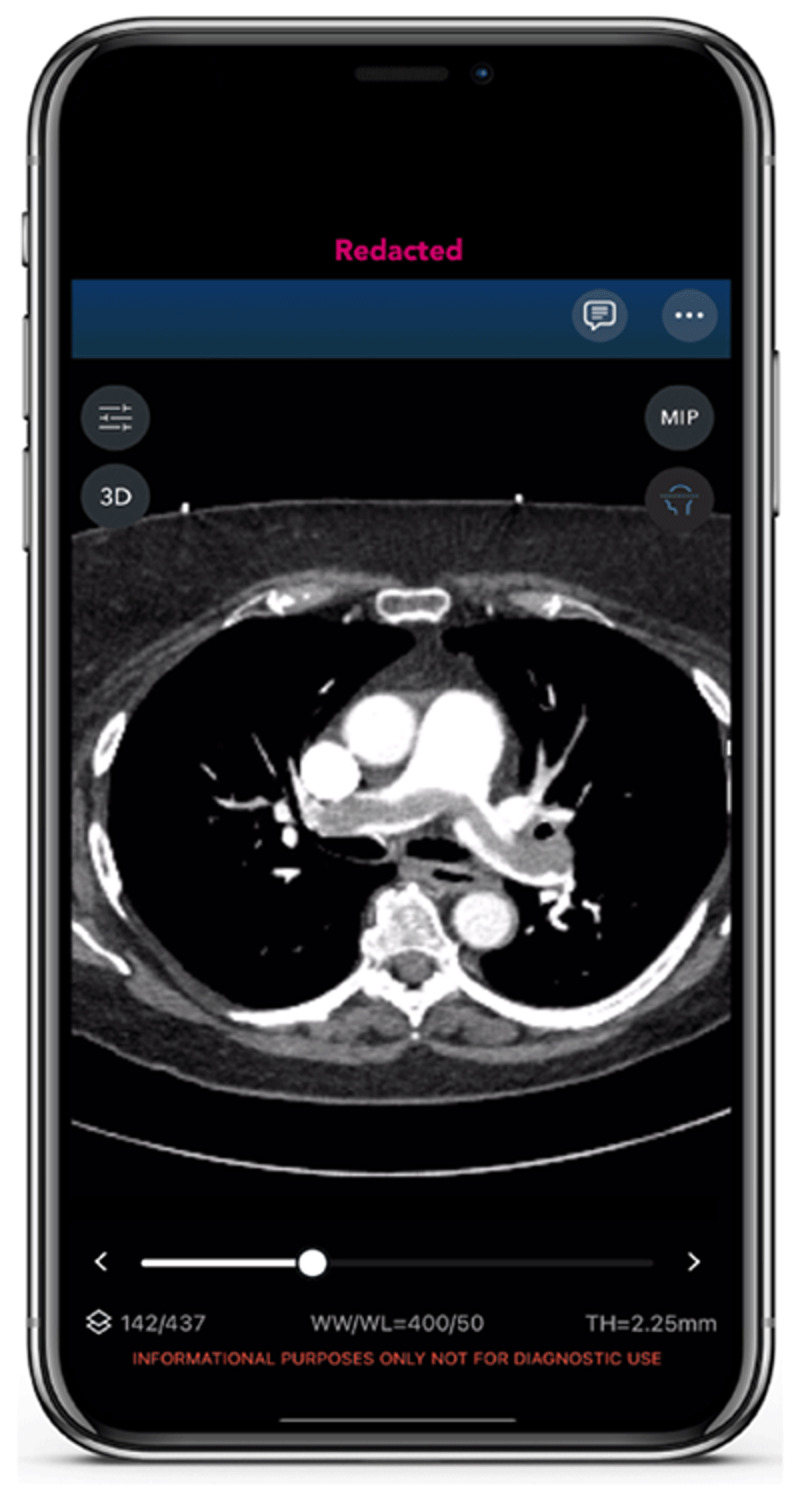
Saddle embolism in Viz.ai app.

Early and automated identification of a PE, accomplished through AI-powered analysis of both images and certain clinical data, enables rapid notification of the institutional PERT. Speedy mobilization driven by these AI solutions can facilitate prompt evaluation and decision-making, potentially improving outcomes and saving lives. To optimize the utility of AI-based detection of PE, companies have developed mobile phone apps to work in conjunction with their AI programs (Figure 1). These apps further enhance communication among medical team members. Practical features of these apps include instantaneous notification of selected team members who are on call, a platform for communication between clinicians, and the capability to share images and other clinical information that complies with the US Health Insurance Portability and Accountability Act privacy laws. Combining early detection, rapid PERT alert, speedy dissemination of information, and a ready communication tool can lead to significant reduction in time-to-treatment. One recent study demonstrated reduction in time-to-procedure from 202 to 55 min after implementing an acute stroke AI program.^2^ Similar reductions can be expected with AI for PE.

### Risk Stratification

Risk stratification is the integration of all data to estimate relative risk of mortality/morbidity. Making sense of the multiple predictors and risk calculators for acute PE—such as the Pulmonary Embolism Severity Index (PESI), simplified PESI (sPESI), PERC (Pulmonary Embolism Rule-out Criteria) rule, Wells criteria, Geneva score, and Hestia criteria—and using them to triage patients appropriately is one of the more controversial and challenging aspects of PE care. The multiple different approaches lead to variability in the care of acute PE. AI programs can integrate all potentially relevant data (even parameters not ordinarily considered to be relevant) and, with application of ML and analysis of treatment and outcomes, inform the “precision” management of individual patients. Ultimately, collection of data and outcomes will lead to better risk stratification tools.

### Determination of Optimal Therapy

Which acute PE patients require escalation of therapy (eg, catheter-based or surgical intervention)? On this issue, there is tremendous variation among practitioners. By utilizing ML to process all available data, AI promises to ultimately identify which patients should have advanced therapy and which advanced therapy is most likely to lead to a good outcome.

### Monitoring Progress During/After Intervention and Establishing Disposition

Certain programs may enable intercalation and ongoing automated analysis of “background” clinical data throughout the entire hospital course. Such programs are already in use for intensive care unit (ICU) patients, helping to determine whether and when care can be safely deescalated and, conversely, when patients are deteriorating and require intervention, such as early intubation. These programs are more accurate, consistent, and timely than physician assessment. Similarly, through ML, AI algorithms can recognize patterns in patients with acute PE that might indicate either expected improvement or worrisome deterioration requiring additional measures. The same ongoing automated analytics may assist in establishing personalized disposition for each patient with PE.

### Ascertaining Risk of Long-Term Consequences

The long-term consequences of acute PE are not well understood. Although the incidence of chronic thromboembolic pulmonary hypertension (CTEPH) is said to be approximately 5%, a substantial percentage of patients develop chronic thromboembolic disease (CTED) and remain partially disabled. Each individual patient’s “PE journey” is unique. That said, by acquiring information regarding the clinical course of tens of thousands of acute PE patients, AI and associated ML can provide insight into each individual’s likelihood of developing longer-term consequences. We also may glean information about the prevention of CTEPH or CTED.

### Expansion of Evidence Base

One of the most important aspects of AI programs is the promise to assimilate data in an ongoing fashion and subsequently coalesce those data to expand the evidence base for PE to inform the field. The resulting data analyses will result in better care standardization and more informed decision-making in the management of PE. The data gleaned will be automatically entered into the PERT Consortium™ PE Registry and used to inform the next wave of guidelines.

## Viz PE Solution Improves Time to Evaluation

Implementation of an AI PE triage platform can have dramatic effects on the time to evaluate a patient with a potential PE. In October of 2022, Viz.ai’s PE Module was implemented at TriHealth Bethesda Hospital, a tertiary referral center in Cincinnati, Ohio. After implementation, a retrospective analysis of patients diagnosed with PE was performed, and patient populations were compared between pre- and postimplementation of the Viz.ai PE Module.^15^

### Statistical Analysis

Descriptive statistics were used for comparisons. Several variables were compared by two-sample t test. All analyses were performed using SPSS (Statistical Package for the Social Sciences, IBM, version 29). Several data points were compared: elapsed time from the initiation of the CPTA scan to the time a radiologist’s report was issued; elapsed time from the initiation of a CTPA scan to the time of consulting a treating physician; elapsed time from CTPA scan initiation to AI alert. The time of PERT activation was also recorded.

In the pre-AI group, 22 patients were evaluated compared to 29 patients in the post-AI implementation group. Time-to-consult in the pre-AI cohort (mean 240.45 min, SD 320.2 min) was significantly longer than in the post-AI cohort (mean 6.72 min, SD 2.7 min); t (49) 3.943; *P* < .001) (Table 2). The difference in time-to-radiology between cases with PERT activation (n 9, mean 29.89 min, SD 21.038 min) and cases without PERT activation (n 20, mean 139.30 min, SD 21.038 min) was dramatically different as well.

**Table 2:** Time to triage. AI: artificial intelligence; PERT: pulmonary embolism response team


COHORT	PRE-AI (n = 22)	POST-AI (n = 29)	POST-AI NON-PERT (n = 20)	POST-AI PERT ACTIVATION (n = 9)

Time-to-consult	240.45 min (*SD =* 320.248)	6.72 min (*SD* 2.671)	–	–

Time-to-rad	30.45 min (*SD* = 22.211)	105.34 min (*SD* = 109.841)	139.30 min (*SD* = 116.9)	29.89 min (*SD* = 21.038)


In the pre-AI population, the time-to-consult ranged from 5 to 1,315 min. In the post-AI cohort, time-to-consult ranged from 2 to 15 min. PERT was initiated notably faster in the post-AI implementation cohort than in the pre-AI cohort. The patient demographics for the population studied are depicted in Table 3.

**Table 3:** Patient and pulmonary embolism characteristics. artificial intelligence; BMI: body mass index; VTE: venous thromboembolism; PE: pulmonary embolism; RV/LV: right ventricular/left ventricular


CHARACTERISTIC	PRE-AI (n = 22)	POST-AI (n = 29)

Sex (% female)	45.4% (10/22)	55.2% (16/29)

Mean age (years)	57.55	67.76

Sedentary lifestyle	54.5% (12/22)	51.7% (15/29)

Recent long-distance travel	13.6% (3/22)	17.2% (5/29)

History of obesity(BMI > 30)	63.6% (14/22)	44.8% (13/29)

Prior VTE	36.4% (8/22)	20.7% (6/29)

Prior cancer diagnosis	13.6% (3/22)	20.7% (6/29)

Recent surgery	22.7% (5/22)	17.2% (5/29)

Prior thrombophilia	9% (2/22)	0% (0/29)

Use of hormone replacement	4.5% (1/22)	6.9% (2/29)

Centrally located PE	86.4% (19/22)	72.4% (21/29)

RV/LV ratio ≥ 1.5	59% (13/22)	48.3% (14/29)

Intervention performed	100% (22/22)	41.4% (12/29)


## Viz PE Solution: Shorter Time-to-assessment and Anticoagulation and Decreased PE Mortality

**Figure 2:**
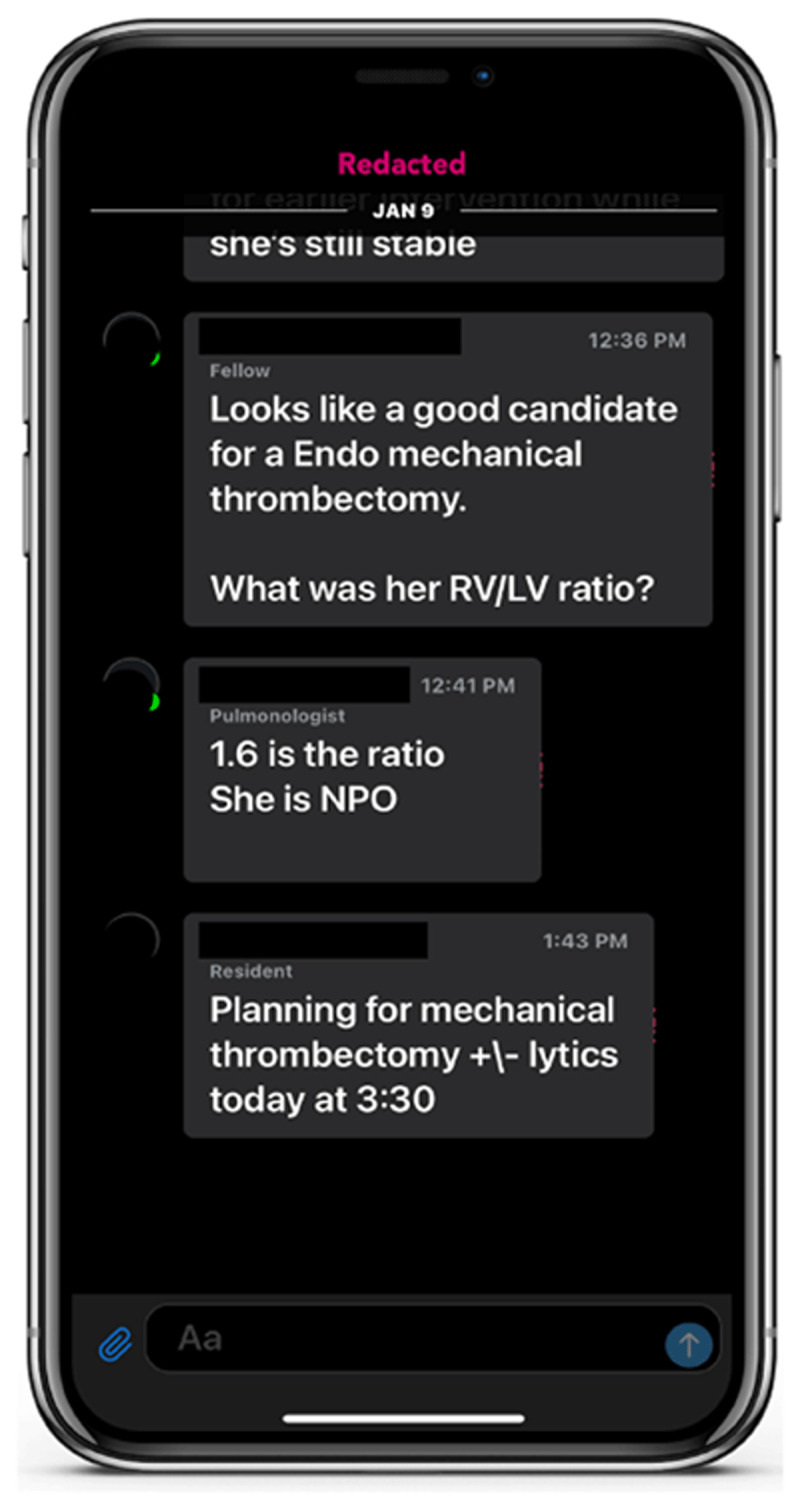
Specialist communication platform.

In October 2022, TriHealth implemented an AI-powered parallel workflow tool designed to automatically detect and triage patients with suspected PE (Figures 1, 2). The aim of this study was to evaluate the clinical impact of AI software on time-to-assessment, time-to-anticoagulation, and patient outcomes.^19^

A review of patients diagnosed with PE between January 2017 and July 2023 (N = 158) was performed and presented at the American Venous Forum’s annual meeting in 2024. Demographics and risk factors were noted (Table 4). Retrospectively collected data on PE patients prior to AI implementation (pre-AI group) was compared against PE patients following AI implementation (post-AI group). Descriptive statistics were used for comparisons. Several variables were compared by two-sample t test. All analyses were performed using SPSS. Collected metrics included scan-to-assessment time, scan-to-alert time (used as a surrogate for scan-to-assessment time following AI implementation assuming best practice), time of anticoagulant administration, PERT activations, and in-hospital mortality.

**Table 4:** Patient characteristics. AI: artificial intelligence; BMI: body mass index; VTE: venous thromboembolism; PE: pulmonary embolism; RV/LV: right ventricular/left ventricular


CHARACTERISTIC	PRE-AI (N = 113)	POST-AI (N = 45)

Sex (% female)	61 (53.9%)	25 (55.5%)

Mean age (years)	60.1	65.3

Sedentary lifestyle	48 (42.4%)	24 (53.3%)

Recent long distance travel	14 (12.3%)	9 (20%)

History of obesity(BMI > 30)	66 (58.4%)	25 (55.5%)

Prior VTE	28 (24.7%)	8 (17.7%)

Prior cancer diagnosis	19 (16.8%)	9 (20%)

Recent surgery	28 (24.7%)	8 (17.7%)

Prior thrombophilia	8 (7.1%)	0 (0%)

Use of hormone replacement	16 (14.1%)	4 (8.8%)

Centrally located PE	96 (86.7%)	35 (77.7%)

RV/LV ratio ≥ 1.5	73 (64.6%)	26 (57.7%)


Scan-to-alert time in the post-AI group (n = 45; mean 5.47; SD 2.76) was significantly faster than scan-to-assessment time in the pre-AI group (n = 113; mean 318.42; SD 339.99; t[156] = 6.163, *P* < .001). Anticoagulants were administered significantly faster for cases with PERT activation (n = 12; mean 83.17; SD 61.32) compared to cases without PERT activation in the post-AI group (n = 24; mean 164.96; SD 82.88; t[34] = –3.021, *P* = .005). In-hospital mortalities decreased from 8.4% (pre-AI) to 2.2% (post-AI). All mortalities took place in cases without PERT activation (Table 5).

**Table 5:** Pre-AI versus post-AI time to assessment. AI: artificial intelligence; PERT: pulmonary embolism response team


	PRE-AI	POST-AI	*P* VALUE

**Time-to-assessment**	318.42 min (SD 339.99)	5.47 min (SD 2.67)	*P* < .001

**In-hospital mortalities**	10 (8.8%)	1 (2.2%)	

	**With PERT**	**Without PERT**	

**Time-to-anticoagulation**	83.17 min (SD 61.32)	164.96 min (SD 82.88)	*P* = .005

**In-hospital mortalities**	10	1	


## Conclusion

Artificial intelligence can provide great benefit in many areas of health care, and nowhere is that benefit more valuable than in the treatment of time-sensitive illnesses like PE. Data show that AI can accelerate the time to decision-to-treat and can further improve intra-service line communication once the decision-to-treat has been reached. Published data evaluating the impact of AI on outcomes suggests a relationship between the faster triage and treatment that AI can help provide with a reduced risk of mortality.

## Key Points

- Pulmonary embolism (PE) is a serious, time-sensitive disease process.
- Challenges exist in the diagnosis, triage, decision-making, and treatment of PE.
- Artificial intelligence (AI) can be a useful tool in expediting patient diagnosis and treatment.
- AI has shown to be valuable in the triage and treatment of other time-sensitive diseases, such as acute ischemic stroke.
